# Gas Sensors Characterization and Multilayer Perceptron (MLP) Hardware Implementation for Gas Identification Using a Field Programmable Gate Array (FPGA)

**DOI:** 10.3390/s130302967

**Published:** 2013-03-01

**Authors:** Fayçal Benrekia, Mokhtar Attari, Mounir Bouhedda

**Affiliations:** 1 Laboratory of Instrumentation (LINS), Faculty of Electronics and Computers, USTHB PO Box 32, Bab Ezzouar 16111, Algiers, Algeria; E-Mail: mattari@usthb.dz; 2 Department of Electrical Engineering and Computers, Faculty of Science and Technology, UYFM 26000, Medea, Algeria; E-Mail: bouhedda.mounir@univ-medea.dz

**Keywords:** e-nose, gas sensor array, pattern recognition, neural network classifier, pic-microcontroller, FPGA-implementation

## Abstract

This paper develops a primitive gas recognition system for discriminating between industrial gas species. The system under investigation consists of an array of eight micro-hotplate-based SnO_2_ thin film gas sensors with different selectivity patterns. The output signals are processed through a signal conditioning and analyzing system. These signals feed a decision-making classifier, which is obtained via a Field Programmable Gate Array (FPGA) with Very High-Speed Integrated Circuit Hardware Description Language. The classifier relies on a multilayer neural network based on a back propagation algorithm with one hidden layer of four neurons and eight neurons at the input and five neurons at the output. The neural network designed after implementation consists of twenty thousand gates. The achieved experimental results seem to show the effectiveness of the proposed classifier, which can discriminate between five industrial gases.

## 1. Introduction

The past decade has seen significant research activity in the development of electronic noses (ENs) for a wide range of applications in civil and military environments. Most of these works have been focused on systems using small size and low cost fabrication microelectronic gas sensors, which makes them attractive for consumer electronic applications [1–3].

Unfortunately, gas sensors present a lack of selectivity and therefore, respond similarly to a wide variety of gases. Thus, the general structure of an EN, which combines an array of sensors with signal pre-processing and pattern recognition algorithms, has been widely accepted and is being used by researchers in this field [4–7].

A number of pattern recognition algorithms have been applied to electronic nose applications such as artificial neural networks (ANNs) with multilayer perceptron (MLP), radial basis functions (RBF), self organizing maps (SOM), K nearest neighbours (KNN), discriminate function and density models [6,8–10]. Recently, many real time and portable smart sensor applications have described the requirements for hardware implementation of pattern recognition algorithms. For this purpose, it is necessary to choose a pattern recognition algorithm with good performance in terms of classification accuracy and acceptable architecture in order to implement an electronic nose. It is also important to find an optimum combination of gas sensor array.

**Figure 1.**
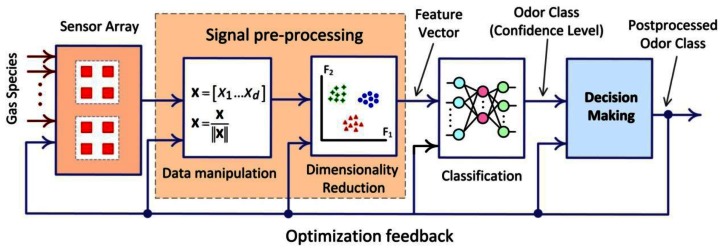
Building blocks of the pattern analysis system for an EN.

As illustrated by Figure 1 [2], the electronic nose system can be divided into five blocs: The sensor array for sensing the different gas species is present in the chamber, Analog and digital signal processing for increasing noise figure (NF) at the level of signals, Feature extraction, Classification and Decision-making are also used.

Actually, the application of artificial neural networks has emerged as a promising area of research in the field of instrumentation and measurement. It provides a neurocomputing approach for solving complex problems and particularly in classification systems. The Field Programmable Gate Array (FPGA) technology allows for developing specific hardware architecture within architectures using a flexible programmable environment. This feature of FPGA gives the designer freedom in the design of the hardware architecture and configuration, contrary to the microprocessors where the architecture is imposed.

Three qualities are targeted overall this work: simplifying the hardware architecture, reducing the cost and power consumption. Firstly, an Artificial Neural Network (ANN) is synthesized with minimizing the number of neurons in the hidden layer. Secondly, the obtained neural architecture is implemented on a FPGA board (XC4020E from Xilinx, Abingdon, Maryland 21009, USA) using Very High-Speed Integrated Circuit Hardware Description Language (VHDL). A microsystem based on a PIC microcontroller is used as an interface between the sensor data and the FPGA classifier.

In this context, a new digital multilayer neural network (DMNN) is proposed to synthesize the classification bloc of the electronic nose system. The result of simulation and experimental tests shows the effectiveness of the realized classifier. The rest of this paper is organized as follows: Section 2 presents the integrated micro-machined gas sensor technology and the experimental characterization of the integrated gas sensor array. Section 3 provides the details of the neural network based on the MLP classifier developed in this study. Section 4 addresses the digital hardware implementation of the neural network and presents the detailed architecture of its building blocks. Finally, Section 5 draws the conclusions and gives an outline of future works.

## 2. Gas Sensors Technology and Experimental Characterization

### 2.1. Gas Sensors Technology

**Figure 2.**
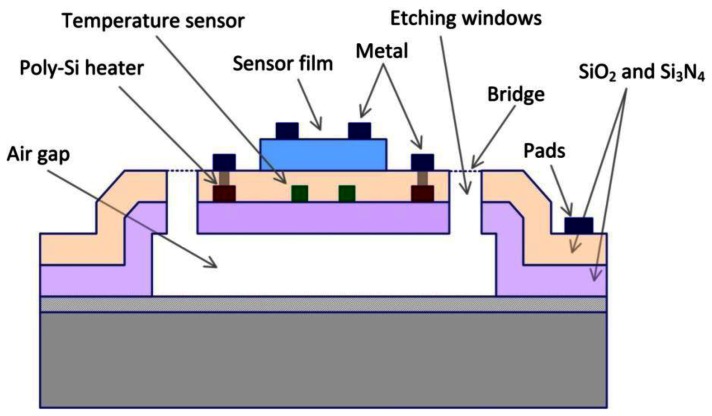
Cross section of the gas sensor in the previous work [12].

Tin oxide is still the most popularly used substance for the detection of combustible gases and toxic contaminants. Microelectronic gas sensors based on tin oxide films are therefore extensively used in gas detection applications. Such devices are sensitive to specific gases when heated at high temperature levels (around 300 °C). To reach such high temperatures, a microstructure called the micro-hotplate (MHP) was developed [11]. This structure is built using either front-side or back-side etch bulk micromachining techniques. The thermally isolated hotplate was fabricated using a surface silicon micromachining technique. The front-side surface machined MHP permits one to retain all the desirable thermal characteristics that are essential to the integrated gas sensor applications. The cross-sectional view of the device is shown in Figure 2.

The MHP is suspended by four micro bridges at the four corners. The bridges are 30 μm wide and 58 μm long. The area outside the MHP remains at the silicon substrate temperature, which reduces the thermal crosstalk between individual MHPs in a sensor array system and when supporting electronic circuitry is to be integrated with the MHP on the same chip. In order to improve the temperature uniformity of the MHP, a polysilicon heater ring is placed at the outer perimeter of the microstructure. The polysilicon resistor at the center monitors the temperature of the MHP. The insulating air-gap was formed by etching away a polysilicon sacrificial layer. Finite element thermal analysis suggested that 1.5–2 μm air-gap provides effective thermal isolation for the MHP.

The device was fabricated using our in-house 4 μm design rule process. The device requires seven masks and occupies an area of 120 × 120 μm. The stability of the resistance in the presence and in the absence of the gas, was experimentally characterized [12]. It was found that the sensor exhibits very good stability (up to 500 cycles) and excellent (as low as 1 ppm) sensitivity to CO. Based on this sensor structure, an integrated gas sensor array, including four individually controllable units was developed.

Each sensor has its own heater and temperature sensor. Three different sensing films are used to implement the sensor array. One sensor (sensor 1) is based on Au/SnO_2_; another sensor (sensor 2) is based on Pt/Cu (0.16 wt%)-SnO_2_, and the remaining two sensors are based on Pt/SnO_2_ (sensors 3 and 4). In total, two chips were used and calibrated by tuning their selectivity to a given set of gases using the temperature parameter. Before carrying out electrical measurements, the temperature of the micro-hotplate is calibrated by first recording the current flow through the heating layer and the temperature (sensor resistance). The sensitivity to CO was found to reach its peak at about 300 °C for sensor 3 and sensor 4 (Pt/SnO_2_), the remaining sensors also respond to variations in the CO concentration, but to a lower extent. A good sensitivity to H_2_ was obtained at about 260 °C, while a good sensitivity to CH_4_ was obtained at about 300 °C. Table 1 summarizes all the sensors used, together with their different parameters.

It should be noticed that the two chips are identical; however, the operating temperature is different, allowing us to tune the selectivity of the two chips to different gases. Using the temperature parameter to set the selectivity of gas sensors is gaining greater interest by researchers in this field, mainly because the selectivity is greatly influenced by the operating temperature [13].

The two chips provide eight responses, which could be seen as a fingerprint or a signature corresponding to a given gas mixture, which can then be exploited by a pattern recognition system in order to built a selective detection systems, as will be described in the next sections.

### 2.2. Experimental Characterization

**Figure 3.**
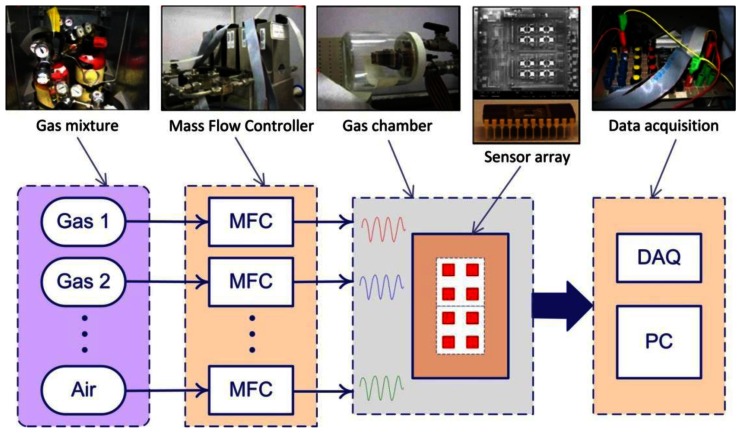
Experimental setup used to characterize the sensors. *V* stands for Valve, *MFC* stands for mass flow controller, and the *DAQ* is the data acquisition board used to control the setup and to acquire the signals from the sensor array [4].

An automated experimental setup was built in order to perform electrical and gas sensing characterization. The setup can be used to measure gas sensing characteristics for well defined temperature cycles and gas concentration levels. Figure 3 illustrates an overall view of the system, including the gas chamber, the gas delivery system as well as the data acquisition system. A gas chamber with a diameter of the 90 mm and a reaction volume of 100 cm^3^ was used for the experiment. The chip carrier is inserted into a chip socket and placed inside the chamber with feed-through wires used for resistance measurement and temperature control.

The gas delivery system includes valves (V) and three mass flow controllers (MFC, with a maximum flow rate of 500 mL·min^−1^) for the tested gas and one MFC for the synthetic air (1 L·min^−1^). A data acquisition board (DAQ) from National Instrument is used in order to control the valves and the MFCs. The DAQ is also used to record the output of the sensors for further processing. The gas concentrations in the sensor chamber are adjusted by selecting the correct flow rate for different gases. Input signals generated by the data acquisition board and used to control the MFC are pulse signals corresponding to different concentrations. The mode of operation is therefore, an online measurement without reference gas with gradually increasing concentrations.

**Figure 4.**
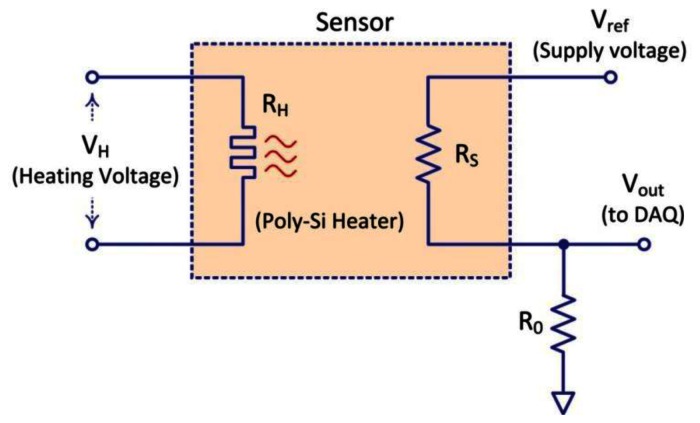
Potentiometric circuit conditioning of the gas sensor.

The temperature of the sensors is constantly monitored by periodically reading data from the integrated temperature sensor. Figure 4 shows the schematic of the circuit used to monitor the sensor response and the operating temperature. Two external voltages are applied to the sensor. One is used to pre-heat (V_H_) the sensor while the second supply (V_sup_) is used to read-out the output voltage through a voltage divider.

**Figure 5.**
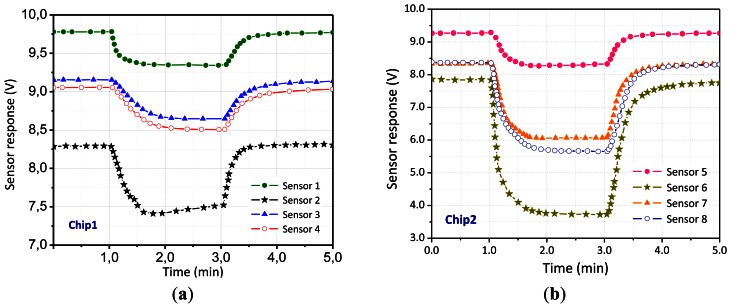
Typical response of the sensors array. The figure on the left shows the response of chip 1, whereas the figure on the right shows the response of chip 2. The voltage measurement of the sensor decrease when exposed to the analysed gases.

The output is then processed by the DAQ. The micro-hotplates of each chip are heated to a particular temperature by flowing the precalibrated current through the heating element. A current flow of 2.8 mA·μm^−2^ through the heating layer is required for an operating temperature of about 300 °C. This corresponds to a maximum power consumption of about 200 mW per chip. Table 2 summarizes the chip features as well as the characterization setup parameters. The sensors output are raw voltage measurements in the form of exponential-like curves typically described by Figure 5. Gases used in the experiment are carbon monoxide (CO), methane (CH_4_), hydrogen (H_2_), and two binary mixtures: one of carbon monoxide and methane (CO–CH_4_) and another of carbon monoxide and hydrogen (CO–H_2_). Vapors were injected into the gas chamber at a flow rate determined by the mass flow controllers (MFC). The steady-state values of the array sensor were recorded while periodically injecting different gases and the baseline response of each sensor was normalized using the Euclidean norm.

Normalization has been previously employed in gas discrimination applications where the identification must be based on signature pattern, and not on the concentration dependent amplitudes [14–16]. On one hand, normalization is useful to set the range of values for sensors’ output to [0,1] range in order to avoid the data pattern with larger signal magnitude to dominate in the data space.

**Figure 6.**
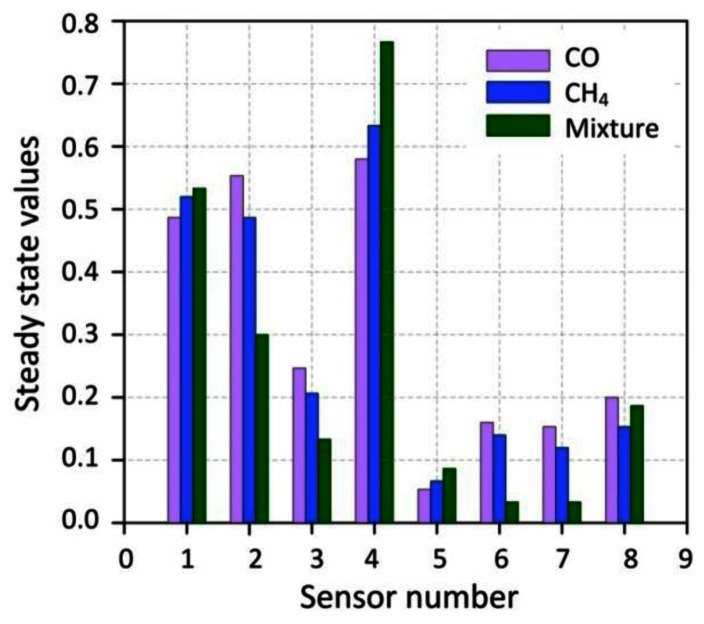
Histograms showing the response patterns of the eight gas sensors exposed to CO, CH_4_, and their mixture.

On the other hand, normalization is applied to remove the concentration dependence within the data space. Because the concentration for an unknown gas is also unknown, the identification must be based on signature pattern, and not on the concentration dependent amplitudes. Figure 6 shows an example of a typical steady-state response for the sensor array exposed to different gases. We can note that the response of the two chips is quite different due to different operating temperatures, as well as mismatch in the fabrication process.

A gas data set of 220 patterns (each pattern consists of eight sensor responses) was created to train the different density classifiers and to evaluate their identification performances.

### 2.3. Classification Results

To evaluate the performance of MLP as a classifier, we performed discrimination experiments on six data-sets which are reported in Table 3. The Breast cancer databases, Hepatitis, Crabs and Iris data-sets are taken from the UCI repository. The last two data-sets are related to gas discrimination and were obtained from our gas sensor experimental setup [4].

The first odor data-set is based on the detection of three gases (CO, H_2_, mixture of CO and H_2_) using seven tin-oxide gas sensors. The second odor data-set is based on the detection of five gases (CO, H_2_, CH_4_, mixture of CO and CH_4_, mixture of CO and H_2_) using eight tin-oxide gas sensors. We compared MLP with a wide range of classification algorithms including K-Nearest Neighbor (KNN), Radial Basis Function (RBF) and Probabilistic Principal Component Analysis (PPCA). Different competing classifiers are built on previously described data-sets. Generalization performances were estimated using a 10-fold cross validation approach. Table 3 reports the classification performance using different classifiers. MLP achieves the best classification performance for the tested data-sets.

## 3. Artificial Neural Network Modeling

One of the most important phases in the design of a supervised ANN is the data collection and data preparation, thus the examples used for training must be representative of all the possibilities concerning the application. Researchers that have used ANNs with supervised learning support the previous statement [17,18]. The steady state values used for this work have been taken from the experimental setup [4]. Where the data-set consists of two matrixes, one matrix for the training patterns: patterns (220 × 8) and another matrix for class labels: labels (220 × 5). This data-set are summarized in Table 4.

### 3.1. Initial Data Analysis

Data analysis consists in dividing data into separate columns, defining type of the columns, filling out missing number values, defining the number of categories for categorical columns, *etc.*

Data analysis revealed the following results:

- Nine columns and 220 rows were analyzed;
- Nine columns and 218 rows were accepted for neural network training;
- Data partition method: random;
- Data partition results: 162 records to Training set (74.31%), 28 records to Validation set (12.84%) and 28 records to Test set (12.84%).

As can be seen, the database is divided into three sets. While the training sets are used only for training, the validation and testing sets are also used for testing.

### 3.2. Data Preprocessing

This is the phase in which the above defined data are prepared and reshaped for an easier use and obtaining best results, according to the requirements and the imposed results. Pre-processing means the modification of the data before it is fed to a neural network (NN). Preprocessing transforms the data to make it suitable for NN (for example, scaling and encoding categories into numeric values, “one-of-n” or binary) and improves the data quality (for example, filtering outliers and approximating missing values), as different software uses different methods [19].

The One-of-N encoding is method of encoding categorical columns into numeric ones. Each new numeric column will represent one category from the categorical column data. (for example the desired outputs were set as follows: if a response was collected in our dataset from carbon monoxide (CO), the value of the desired output corresponding to CO was set to 1 and the rest of the outputs were set to 0 *i.e.*, (10000)). In Table 5, the characteristics of preprocessing data are shown.

The results of completed preprocessing process are:

- Columns before preprocessing: 9;
- Columns after preprocessing: 13;
- Input columns scaling range: [−1..1];
- Output column (s) scaling range: [0..1];
- Numeric columns scaling parameters: (see Table 5).
- Categorical column encoding parameters:
- Class: One-of-5.

### 3.3. Artificial Neural Network Structure

After building and testing several ANN with feed forward structures, having in mind the comparison of the errors between the real data and ANN output data, the best ANN network was defined.

**Figure 7.**
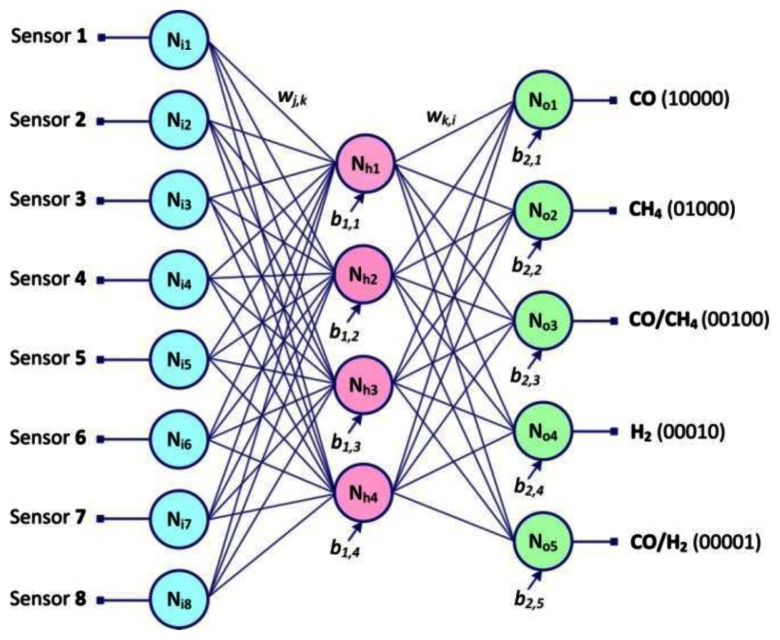
Simplified graphic representation of ANN with structure 8-4-5.

This has the structure shown in Figure 7: Eight neurons in the input layer, four neurons in the hidden layer and five neurons in the output layer. These neurons are fully interconnected to each other between adjacent layers.

### 3.4. Training

**Figure 8.**
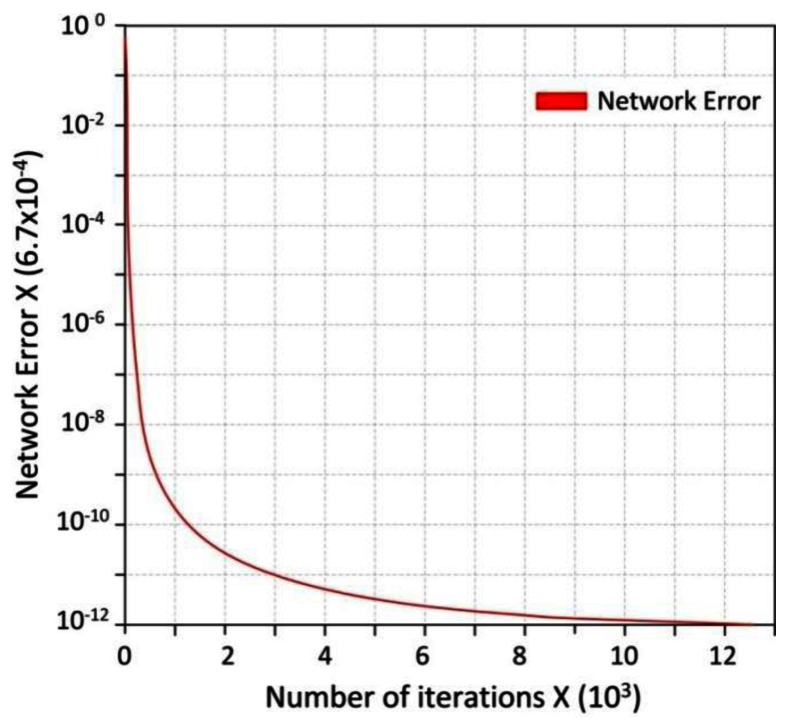
Evolution of the network errors.

Being an essential phase in the use of ANN, the training must use certain training algorithms which essentially modifies the structural elements of ANN (weights) modified through several iterations. Those modifications establish the future ANN accuracy. For the selected ANN, the most common training algorithm is the Back Propagation (BP) algorithm. Back propagation is the best-known training algorithm for multilayer neural networks. It defines the rules of the propagation of the network error back from the network output to network input units and adjustment of network weights along with this BP. Training is done by off-line simulation of the network on a PC. The final values of weights are obtained at the end of the training session. The input layer has been added for buffering the data. Figure 8 shows the evolution of recognition rate with the number of iterations. After 13,001 iterations, the results of the ANN test are summarized in the Table 6.

## 4. Hardware Implementation

### 4.1. Digital Implementation of the NN

#### 4.1.1. Data Representation

A number's format (fixed, floating point) must be considered for the inputs, weights and activation function [7]. The resolution (number of bits) should also be considered. Increasing the precision of the design elements significantly increases the resources used. Accuracy has a great impact in the learning phase; so the number's precision must be as high as possible during training. However during the learning phase, lower precisions are acceptable. The resulting errors will be small enough to be neglected, especially in classification applications. The neurons inputs are encoded in 11 bits (three bits for the integer part and eight bits for the fractional part), because the input neurons are normalized between −1 and 1, 8-bit fractional part gives a precision of 1/256 which is sufficient. For the weights and biases, the numbers are coded on 16 bits (eight bits for the integer part, eight bits for the fractional part). To represent negative numbers, the 2's complement method is used.

#### 4.1.2. Hidden Layer of the NN

**Figure 9.**
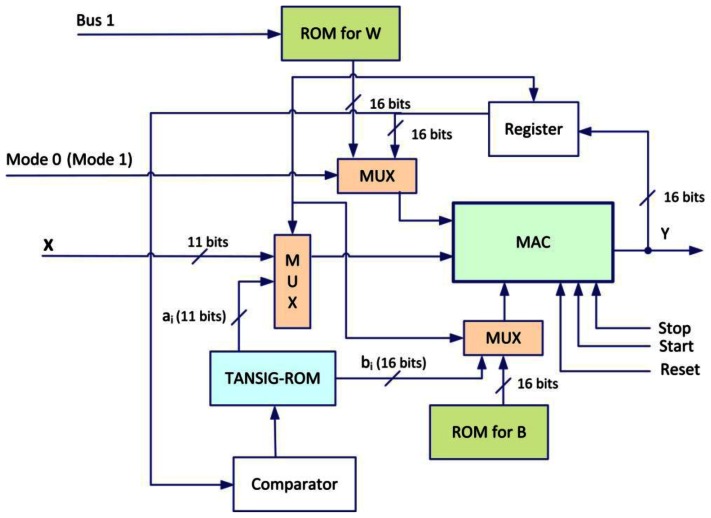
Schematic of digital neuron (hidden unit).

Figure 9 shows the schematic structure of the designed neuron of the hidden layer which contains three 2-to-1 multiplexers, three ROMs (ROM for Weights, a ROM for Biases and a ROM for *a_i_* and *b_i_*; *a_i_*, *b_i_* represents the parameters of the segments for linear functions), MAC (Multiplier Accumulator Component), a register and a comparator.

The neuron has two operating Modes (Mode 0 and Mode 1). For the Mode 0, the neuron carries out the pondered sum of the entries by their synaptic weights using MAC. In Mode 1, it carries out the approximation by segments of the sigmoid function using a comparator with the same MAC used in the Mode 0. The use of 2-to-1 multiplexer simplifies commutation between the two modes, and the control signals are generated by an external sequencer which ensures synchronization between the various processes.

#### 4.1.3. The MAC

**Figure 10.**
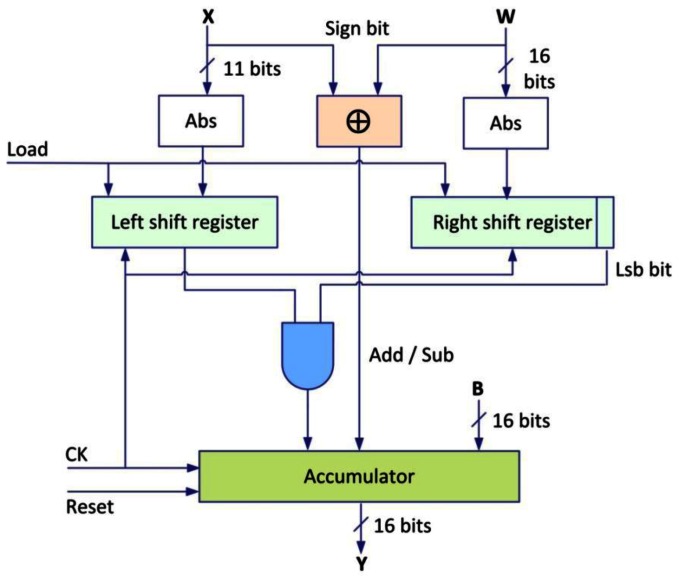
The designed MAC structure.

Figure 10 shows a designed structure of the Multiplier Accumulator Component (MAC), which is composed by the 2's complement and one comparator for the sign, two shift registers (right and left), a network of AND gates and an accumulator.

The 2's complement will use the values of *W* (16 bits) and *X* (input value with 11 bits) which will be complemented if the value is negative, before putting them in the two shift registers, the latter have the function of shifting the bits after each rising edge of the signal clock in the two different directions according to the register, the value of the register on the left is multiplied by the first LSB bit of the right shift register using a network of AND gates.

After initializing by *B* value, the result is stored in the accumulator (ACC) positively if *X* and *W* have the same sign and negatively if *W* and *X* have different sign. This process is repeated 16 times to calculate the product *WX*, and finally the total process is repeated for all the values of *X* and *W* without resetting the contents of the accumulator, to evaluate the Equation (1), finally:

(1) $$Y=B+\sum W_{i}X_{i}$$

#### 4.1.4. Activation Function

**Figure 11.**
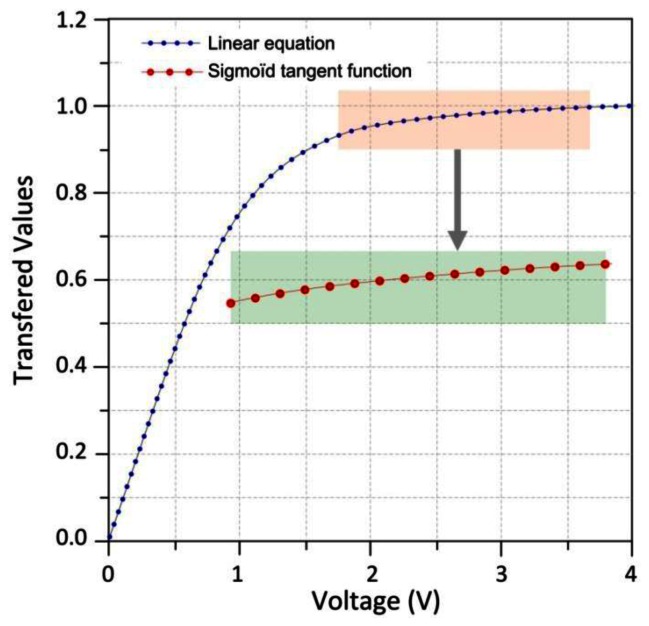
Comparison of sigmoid tangent and linear functions.

For approximating the sigmoid tangent function, a look-up table (LUT) is used. This method has a merit that has a simple structure, that is, it stores output for each input as an address, but has the demerit that takes up a lot of address area to ensure high precision [8]. To solve the problems, the sigmoid tangent function using discontinued linear functions is used in this work. Equation (2) defines the sigmoid tangent function:

(2) $$y=\frac{1-\exp^{-\lambda x}}{1+\exp^{-\lambda x}}=\tan\text{sig}(x)$$

where λ represents the slope of the sigmoid tangent (λ = 1). This system computes the output *y* as ranges of input *x* using sigmoid tangent function. The sigmoid tangent function (tansig) is segmented with 32 segments. Each segment *i* is calculated by:

(3) $$Y^{\prime }=a_{i}X+b_{i}$$

where a_i_, b_i_ represent the parameters of the segments. The segments of the negative and positive part are symmetrical compared to the origin for which only the segments of the positive part is calculated by Equation (3) and the negative part is calculated by symmetry from the positive part. Figure 11 shows the discritization of a continuous sigmoid in several segments.

#### 4.1.5. Output Layer

**Figure 12.**
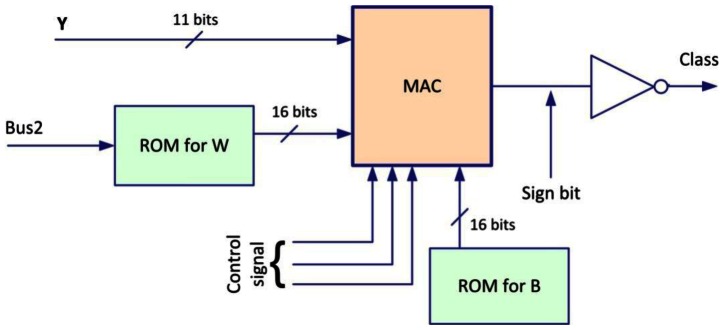
Structure of the output layer neuron.

Figure 12 shows the structure of neurons used in the output layer which contains two ROMs (one for W and the other for the B), a MAC and a converter. The outputs neurons includes only one operating mode, in this mode, the MAC receives the multiplexed outputs of the hidden layer neurons, and realize the pondered sum with the synaptic weights stored in a ROM.

The threshold activation function is fulfilled by a simple converter of sign bit (threshold function has a binary output), and the control signals are generated by an external controller that ensures synchronization.

### 4.2. Electronic Nose Design and Test

#### 4.2.1. Design Description

The electronic nose consists of data acquisition, signal preprocessing, and pattern recognition stages [9]. Our proposed Digital Multi-layer Neural Network (DMNN) can achieve over 93% classification performance. However, the implementation of the whole module will take very large hardware resources. Thus, using a full-custom IC design will lead to high fabrication costs. In addition, the response of the sensor is very slow (the time to achieve steady-state ranges from around a few hundred seconds to a few minutes) [10], thus, full-custom IC design, which is generally used to achieve high-speed operation, is not necessary for our application. Semi-custom designs such as DSP, microcontroller, or FPGA is another viable option, but only if sufficient hardware resources are available.

Due to the slow response of the sensor array, the time constraint is relaxed allowing each stage of the module to operate sequentially. For the whole module, we can first implement data acquisition and signal preprocessing stage on the PIC microcontroller (16F877A) to generate a valid pattern to be processed at a later stage by the pattern recognition module. After signal preprocessing is performed, the gas pattern (Voltage) will be stored in the PIC memory (Flash EEPROM (8K)) and the FPGA will be automatically reconfigured to implement the Digital Multi-layer Neural Network classifier.

**Figure 13.**
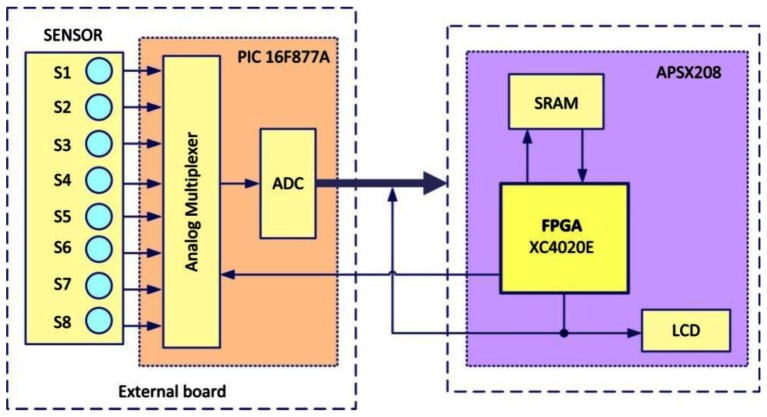
The gas identification module platform.

The DMNN will first read out the voltage values stored previously in the memory of the PIC and perform the classification. The module platform is shown in Figure 13 which consists of two boards: the sensor interface board and the FPGA board. To emulate the response of the sensors, eight variable resistors were used to provide eight different voltage that will correspond to eight sensors outputs, PIC microcontroller 16F877A (contains an analog multiplexer, and ADC (10 bits)) and LCD for displaying combustible gas recognition are located on the sensor interface board, which is used to sample the responses of the sensor array and convert them into digital data.

**Figure 14.**
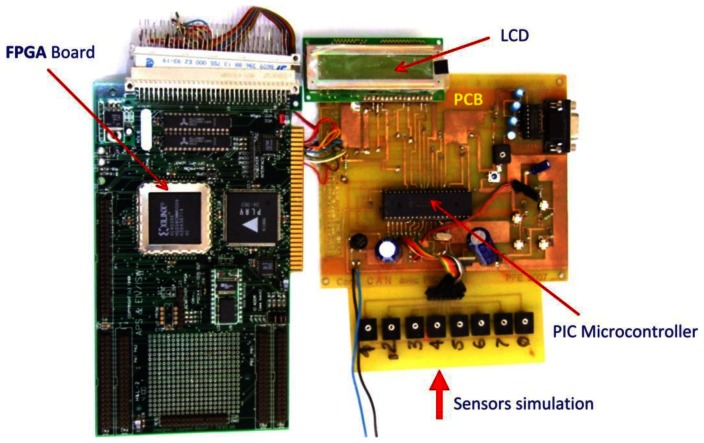
Photograph of the gas identification module board.

The gas identification module is developed with the X208 FPGA platform provided by APS. The FPGA on X208 belongs to Xilinx XC4000E family, which is shown in Figure 14.

#### 4.2.2. Design Flow and Tools

Actually, the synthesis tools allow the use of FPGA resources with schematics entrance as well as an algorithmic one. The algorithmic design is written with VHDL. This language permits the design of complex circuits with structural description or a behavioral one. The synthesis software leads from a VHDL program after compilation to a Xilinx Netlist Format (XNF) file, and the corresponding schematics by means of primitive and X-blocs components with a logic optimization. The functional simulation insures the design functionality before the routing phase of the FPGA.

#### 4.2.3. Test Results

The programmable logic device of SRAM-based FPGA chip (XC4020Ehq208-4) supplied by Xilinx Corporation is used for this system. The Modelsim, Synplify Pro, and Xilinx foundation softwares were used for VHDL programming, simulation, synthesis and chip implementation. The results as shown in Table 7 illustrate the utilization of the device.

**Figure 15.**
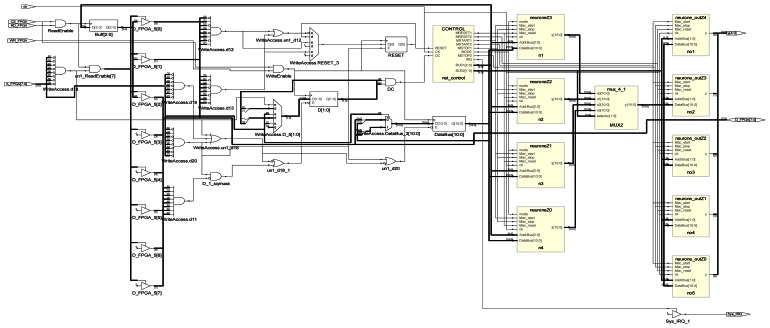
Synthesis result of the digital multilayer neural network.

After simulation and synthesis (as shown in Figure 15) of the whole network, the placement of blocs is taken into account which consists in attaching calculus blocs to the logical operators, choosing the input-outputs pins and routing which consists in creating the necessary interconnections.

The last step is generating a settings file named rawbit with (.rbt, extension). This file in ASCII code contains the information provided to the component FPGA Xilinx, so that it accepts the desired configuration. A clock frequency of 10 MHz is applied to the system, which is the maximum frequency available on X208 APS board, the implementation results and comparisons with different methods available in the related literature are summarized in Table 8.

## 5. Conclusions

In this paper, a primitive digital multilayer neural network (DMNN) with input, hidden and output layers of neurons (8-4-5) is designed and implemented on FPGA XC4020E chip by APS (Associated Professional Systems, Abingdon, Maryland 21009, USA) X208 FPGA test board. The slowness of gas sensor in terms of time response (in the range of few seconds) permits the computation requirements into different blocs. The chip based neural network is designed for a primitive industrial gas discrimination system. The simulation and experimental results of the designed neural network chip shows the effectiveness of the proposed gas recognition system using the DMNN chip in the classification to evaluate the limited gas species of industrial gases. The system can be operating at a frequency of 10 MHz. The reconfiguration time of the FPGA chip is around 26 ns, which can be neglected compared with the speed of the gas sensors (in the range of a few seconds). Future work will include a combining multiple classifiers (such as neural networks or decision trees) to build an ensemble is constitutes an advanced pattern recognition technique for classification accuracy.

## Figures and Tables

**Table 1.** Sensor description.

	**Sensor Number**			
Chip 1 (260 °C)	S1	S2	S3	S4
Chip 2 (300 °C)	S5	S6	S7	S8
Material used	Au/SnO_2_	Pt/Cu-SnO_2_	Pt/SnO_2_	

**Table 2.** Sensor array characteristics and characterization setup parameters.

**Sensor Array Chip’ Features**
Process In-house 4 μm
Sensor array 2 × 2
Power 200 mW at 300 °C
Sensitivity 14 Ω at 55 ppm CO
Response time 1–3 min at 300 °C, 50 ppm CO
Heater resistance 12 KΩ at 300 °C
**Characterization Setup Parameters**

Tested gases CO, CH_4_, H_2_,
Concentrations 25–200, 500–4,000, 500–2,000
Gas chamber D = 90 mm & V = 100 cm^3^
Max gas flow Max 500 mL·min^−1^
Synth. Air flow 1 L·min^−1^

**Table 3.** Classification performance of different classifiers based on the six data-sets. 10-fold cross validation approach is used to evaluate the classification accuracy (in %). *d* and *c* stands for the number of variables and classes respectively.

**Data-Sets (*d*, *c*)**	**MLP**	**Performance (%) KNN**	**RBF**	**PPCA**
Breast (4, 2)	91.36	92.30	92.27	**92.73**
Hepatitis (19, 2)	**83.75**	81.25	**83.75**	75
Crabs (5, 2)	**100**	96.50	**100**	99.50
Iris (4, 3)	**98**	96	95.33	**98**
Odor 1 (7, 3)	**100**	98.75	96.25	97.50
Odor 2 (8, 5)	**93.75**	92.50	86.80	84.10

**Table 4.** The database.

**8 Inputs Patterns 5 Outputs Class Labels (Desired)**											
	**Sensor-1**	**Sensor-2**	**Sensor-3**	…	**Sensor-7**	**Sensor-8**	**CO**	**CH_4_**	**CO-CH_4_**	**H_2_**	**CO-H_2_**
1	−0.48341	−0.32601	−0.12765	…	−0.092448	−0.12887	0	0	1	0	0
2	−0.28166	−0.32896	−0.32135	…	−0.46348	−0.41435	0	0	0	0	1
3	6-0.48962	−0.32074	−0.13708	…	−0.11217	−0.23386	1	0	0	0	0
4	−0.48219	−0.3134	−0.1224	…	−0.08816	−0.13044	0	0	1	0	0
5	−0.49745	−0.212272	−0.071727	…	−0.056235	−0.097484	0	1	0	0	0
…				…	… … … … … … …						
218	−0.52089	−0.32247	−0.15158	…	−0.13233	−0.20177	1	0	0	0	0
219	−0.49159	−0.32963	−0.13762	…	−0.12843	−0.16473	0	0	1	0	0
220	−0.30569	−0.36646	−0.33959	…	−0.27121	−0.59245	0	0	0	0	1

**Table 5.** The preprocessing database.

**Parameter**	**Scaling Range**	**Min**	**Max**	**Mean**	**Standard Deviation**	**Scaling Factor**
Sensor1		−0.54244	−0.14692	−0.454248	0.0915	5.056634
Sensor2		−0.53375	−0.18272	−0.352008	0.07431	5.697519
Sensor3		−0.42172	−0.062699	−0.191719	0.086866	5.570705
Sensor4	[−1..1]	−0.83832	−0.28273	−0.635723	0.168284	3.599777
Sensor5		−0.092242	−0.029856	−0.06805	0.01865	32.058475
Sensor6		−0.51245	−0.042622	−0.190384	0.124915	4.256877
Sensor7		−0.5195	−0.038313	−0.182026	0.122465	4.156388
Sensor8		−0.6414	−0.023022	−0.232022	0.142461	3.238311
Class	[0..1]	0	1	n/a	n/a	n/a

**Table 6.** Results of the ANN test.

**Rate**	**%**
Recognizing in the learning phase	92.07
Recognizing in the validation phase	100
Recognizing in the test phase	89.28
Badly classified patterns	0.45
Not classified patterns	6.81

**Table 7.** Results of synthesis architecture.

Number of CLBs	760 out of 784	96%
CLB Flip Flops	584	-
Four input LUTs	1356 (9 used as route-throughs)	-
Three input LUTs	200 (13 used as route-throughs)	-
16X1 RAMs	86	-
Number of bonded IOBs	26 out of 160	16%
IOB Flops	11	-
IOB Latches	0	-
Number of clock IOB pads	1 out of 8	12%
Number of primary CLKs	1 out of 4	25%
Number of secondary CLKs	3 out of 4	75%
Total equivalent gate count for design	19446	-
Additional JTAG gate count for IOBs	1248	-
Additional JTAG gate count for IOBs	1248	-
Min. period (ns): 100; Max. freq. (MHZ)	10	-
The Average Connection Delay for this design is	6.466 ns (155 MHz)	-
The Maximum Pin Delay	31.460 ns (32 MHz)	-
The Average Connection Delay on the 10 Worst Nets	26.465 ns (38 MHz)	-

**Table 8.** Implementation results on Xilinx Virtex II and XC4020E FPGA chip. Committee machine stands of MLP, RBF, KNN, GMM and PPCA classifiers.

	**Committee Machine [20]** **(Virtex II)**	**This Work (MLP)** **(XC4020E)**
No. of slices and CLB (% of resources)	12,146 (84%)	**760 (96%)**
No. of 4 input LUT (% resources)	20,115 (70%)	**1,356 (86%)**
Frequency (MHz)	50	**10**
Reconfiguration times (ms)	26.26	**26.46 10^−6^**
